# Ohio State Dental Examining Board

**Published:** 1893-01

**Authors:** 


### EDITORIAL.

### Ohio State Dental Examining Board.

This Board, appointed by the Governor, under the recently enacted Dental law, consisting of Drs. Grant Mitchell, of Canton, Grant Mollyneaux, of Cincinnati, L. E. Custer, of Dayton, C. L. Smith, of Columbus, and J. E. Silcott, of Washington C. H., proceeded immediately after their appointment to effect an organization, which was done by selecting Dr. Grant Mitchell as Chairman, and Dr. Grant Mollyneaux as Secretary.

This Board proceeded immediately to the accomplishment of the work assigned to it by the law, which for the first two or three months was very laborious. Notice of the requirements of the law was at once sent to all dentists throughout the State, with the request that they comply with its requirements.

How well this request was regarded may be inferred from the following facts, viz : that up to the present time nine hundred and twenty-one, of whom one-half are college graduates, have registered. So far as the Board is aware, only about twenty-five in the entire State have not as yet registered. There have been examinations of seventeen persons, ten of whom passed and were registered; seven were refused registration. Some who were not prepared for examination and desired to practice, left for other and more congenial fields than Ohio, and a few abandoned practice—at least till they should better prepare themselves for it. We can assure the Board that its labors are duly appreciated by the profession.

